# Barriers and enablers to leadership advancement for women with disabilities: a systematic literature review

**DOI:** 10.3389/fresc.2026.1813535

**Published:** 2026-06-15

**Authors:** Udeme Samuel Jacob, Sri Nurhayati, Mbulaheni Obert Maguvhe

**Affiliations:** 1 Postdoctoral Research Fellow, Department of Inclusive Education, School of Educational Studies, College of Education, University of South Africa, Pretoria, South Africa; 2 Department of Community Education, Postgraduate Program, Institut Keguruan dan Ilmu Pendidikan Siliwangi, Cimahi, Indonesia; 3 Department of Inclusive Education, School of Educational Studies, College of Education, University of South Africa, Pretoria, South Africa

**Keywords:** barriers and enablers, inclusive leadership, intersectionality, leadership advancement, women with disabilities

## Abstract

**Background:**

Women with disabilities are underrepresented in leadership roles despite global commitments to diversity. This systematic literature review examines the barriers and enablers impacting their advancement in these positions, addressing a critical gap at the intersection of gender, disability, and leadership research.

**Methods:**

In accordance with the PRISMA 2020 reporting standards, a structured search was conducted in Scopus, **supplemented by Web of Science and Google Scholar to enhance interdisciplinary coverage**, identifying peer-reviewed studies published between 2021 and 2025. **The timeframe was selected to capture post-pandemic shifts in organisational practices and inclusion discourse.** After screening and eligibility assessment, 20 studies were included in the thematic synthesis.

**Results:**

The findings indicate that barriers to leadership advancement are largely systemic, including inaccessible governance structures, ableist organisational cultures, credibility discounting, restricted autonomy, and gendered care burdens. **These patterns suggest that leadership exclusion is shaped by multi-level structural and relational constraints rather than individual capability alone.** These barriers are further influenced by intersectional factors such as race and socioeconomic status. In contrast, enabling conditions include inclusive leadership practices, openness to feedback, rights-based governance, supported decision-making, and capacity-building through mentoring and development.

**Conclusion:**

The review conceptualises leadership advancement as a multi-level process shaped by structural, organisational, interpersonal, and identity-related dynamics. **Evidence across studies suggests that leadership trajectories may be cumulative, with early constraints influencing later access to leadership opportunities.** By mapping existing evidence and highlighting critical gaps, this review underscores the need for leadership frameworks and policies that recognise women with disabilities as legitimate leaders. **These conclusions should be interpreted in light of the relatively small and heterogeneous evidence base.** Advancing inclusive leadership ecosystems remains essential for reducing entrenched inequities and fostering more representative systems of governance.

## Introduction

Women with disabilities represent a substantial yet marginalised segment of the global workforce. Although disability affects approximately one billion people worldwide, women with disabilities experience compounded disadvantage associated with the intersection of gender and disability (1). These intersecting inequalities are reflected in persistent disparities in employment outcomes, including lower labour force participation, limited access to career progression opportunities, and continued underrepresentation in managerial, professional, and decision-making roles when compared with both men with disabilities and women without disabilities (1–3). Within this study, leadership advancement is conceptualised not only as movement into formal leadership positions but also as access to influence, governance, and decision-making spaces within organisational and institutional settings. This broader conceptualisation reflects contemporary perspectives that view leadership as relational, distributed, and embedded within structural conditions (4, 5).

Research on women's leadership has expanded considerably over the past two decades, offering insights into gendered barriers across sectors such as healthcare, higher education, and corporate environments. Evidence consistently shows that women remain underrepresented in senior leadership roles despite their strong workforce participation (6–9). These disparities are shaped not only by individual-level factors but also by organisational cultures, institutional structures, and leadership norms that privilege dominant forms of authority and marginalise those who do not fit them (10, 11). In many cases, these norms reflect implicit expectations about competence, independence, and uninterrupted career trajectories, which may disadvantage individuals whose experiences do not align with these ideals (12). However, despite the depth of gender-focused research, there remains limited insight into how disability intersects with gender and other social identities to shape leadership pathways (2, 10).

For women with disabilities, these exclusions are often intensified through intersectional dynamics involving race, class, geography, and caregiving responsibilities. Intersectionality provides a critical lens for understanding how multiple forms of disadvantage interact to produce unique experiences of marginalisation (13, 14). In this context, barriers are the structural, institutional, cultural, and interpersonal conditions that constrain leadership pathways, while enablers are the supports and conditions that facilitate participation, recognition, and progression. Empirical evidence highlights workplace discrimination, inaccessible environments, and ableist assumptions as key constraints on advancement (1, 2). These challenges are further reinforced by sociocultural beliefs that position disability as incompatible with leadership, thereby limiting credibility, visibility, and access to authority (11, 15).

Beyond immediate workplace conditions, leadership exclusion is shaped by broader systemic and life-course factors. Restricted access to education, skills development, and professional training limits the accumulation of human and social capital necessary for leadership roles (3, 16). These early disadvantages often translate into reduced confidence, limited professional networks, and constrained access to decision-making opportunities later in life. Intersectional analyses further demonstrate that these disadvantages accumulate across the life course, influencing leadership trajectories over time rather than occurring at isolated career stages (17, 18). This cumulative perspective suggests that leadership exclusion is structurally embedded and temporally sustained, rather than episodic or context specific.

Organisational and structural barriers further constrain leadership advancement. Inaccessible work environments, insufficient accommodations, and limited implementation of inclusive policies restrict participation and progression (2, 19). In addition, limited access to professional networks, mentorship, and sponsorship opportunities significantly reduces opportunities for career advancement, given their central role in leadership development and visibility (4, 20). Persistent inequalities such as wage disparities, work-life conflict, and underrepresentation in leadership positions further reinforce these barriers, creating systemic constraints on progression (12, 21). These challenges are compounded by the absence of targeted leadership development programmes and limited institutional commitment to inclusive governance structures (5).

Despite these barriers, emerging evidence highlights several enabling conditions that can support leadership development and progression. Inclusive organisational practices, including transparent promotion systems, flexible work arrangements, and accessibility reforms, have been shown to enhance participation and reduce structural constraints (7, 19). Mentorship and professional networks play a particularly important role, providing access to guidance, role models, and career opportunities (6, 20). Peer support networks and community-based connections further contribute to building resilience, legitimacy, and leadership identity among women with disabilities (5, 15). In addition, tailored leadership development initiatives, including specialised training programmes and entrepreneurship education, have been identified as effective mechanisms for enhancing confidence, visibility, and career mobility (16, 21).

Individual-level factors also play a role in leadership advancement, although they operate within broader structural constraints. Studies indicate that women with disabilities often demonstrate resilience, adaptive coping strategies, and strong self-management skills to navigate barriers and sustain career progression (22). Emotional intelligence, persistence, and strategic career planning have been identified as key personal resources that support leadership development (4). However, these individual strategies should not be interpreted as substitutes for structural change; rather, they reflect adaptive responses to systemic inequality.

Although literature provides valuable insights into both barriers and enablers, it remains fragmented across disciplines and contexts. Existing studies frequently examine related domains such as employment, governance participation, organisational culture, accessibility, or institutional inclusion without explicitly focusing on leadership advancement. Moreover, while some reviews explore women with disabilities more broadly, they do not isolate leadership pathways, and studies on women's leadership rarely integrate disability as a central analytical dimension (1, 2, 23). This fragmentation limits the development of a coherent understanding of how leadership pathways are shaped across structural, organisational, and life-course dimensions.

In addition, the conceptualisation of leadership varies across studies, with some focusing on formal roles and others examining influence, participation, or decision-making authority. This diversity complicates synthesis and highlights the need for a systematic approach that integrates multiple perspectives. A comprehensive synthesis is therefore necessary to map existing evidence, identify consistent patterns, and clarify the mechanisms through which leadership pathways are constrained or enabled.

Accordingly, this PRISMA 2020-guided systematic review employing thematic synthesis aims to synthesise existing evidence on the barriers and enablers influencing the advancement of women with disabilities in leadership. The study specifically seeks to: (1) identify structural, institutional, and socio-cultural barriers that constrain access to leadership and decision-making spaces; (2) examine organisational, relational, and autonomy-supportive enablers that facilitate participation, agency, and progression; (3) analyse how intersectional and life-course factors shape leadership opportunities and trajectories; and (4) map the distribution and conceptual focus of existing evidence across contexts to identify gaps for future research, policy, and practice.

## Theoretical framework

### Intersectionality theory

Intersectionality Theory was advanced by Kimberlé Crenshaw, who introduced the term *intersectionality* in 1989 to explain how multiple systems of oppression interact to shape social experiences (13, 24, 25). Crenshaw developed the concept through her critique of legal and institutional frameworks that treated discrimination as a single-category issue, particularly in relation to race or gender separately. She argued that such approaches failed to capture the realities of individuals who experience overlapping forms of marginalisation simultaneously, especially Black women whose experiences could not be fully understood through either racism or sexism alone (26, 27). Her contribution transformed critical social theory by demonstrating that inequality operates through interconnected structures rather than isolated categories.

The central proposition of Intersectionality Theory is that social identities such as gender, disability, race, class, sexuality, and age do not function independently. Instead, they intersect within broader systems of power to produce unique patterns of privilege and disadvantage (28, 29). This means that an individual may encounter barriers not simply because they are a woman or because they have a disability, but because these identities combine in ways that create distinct social experiences. The theory therefore rejects single-axis explanations of inequality and calls for more nuanced analyses of how institutions, policies, and cultural norms reproduce exclusion (30).

A major assumption of the theory is that systems of oppression, including sexism, ableism, racism, and class inequality, are structurally linked. These systems reinforce one another through organisational practices, stereotypes, and unequal access to resources. For example, workplace recruitment and promotion processes may appear neutral, yet they can disadvantage women with disabilities when leadership norms are based on masculine ideals of authority and able-bodied assumptions of productivity. Intersectionality Theory helps reveal how such barriers are embedded in institutional arrangements rather than arising solely from individual prejudice (26, 31).

The theory is highly relevant to studies examining barriers and enablers affecting women with disabilities. It provides a framework for understanding why this group may face exclusion from leadership pipelines, limited mentoring opportunities, inaccessible work environments, and underrepresentation in decision-making roles. At the same time, it also explains how enabling factors such as inclusive policies, targeted leadership development, mentorship opportunities, flexible work arrangements, and accessible organisational cultures can reduce structural disadvantages (32, 33). By focusing on the interaction of identities and systems, the theory offers a richer explanation of both obstacles and opportunities.

Beyond its origins in legal scholarship, Intersectionality Theory has become influential across sociology, education, psychology, public health, and organisational studies. Researchers now use it to examine how complex identities shape outcomes in employment, wellbeing, education, and leadership (34, 35). Its enduring significance lies in its ability to move analysis beyond simplistic categories toward a deeper understanding of inequality and social change. For studies on leadership advancement among women with disabilities, Intersectionality Theory provides a strong conceptual foundation for analysing how overlapping identities shape both barriers and pathways to success.

### Social role theory

Social Role Theory was developed by Alice Eagly to explain how gender differences and similarities in behaviour emerge from the social roles assigned to men and women rather than from biology alone (36, 37). The theory argues that societies distribute responsibilities, occupations, and expectations along gendered lines, and these role assignments shape beliefs about how men and women are expected to behave and perform. Thus, many observed behavioural differences reflect social structure rather than fixed personal traits.

The central proposition of Social Role Theory is that the division of labour creates stereotypical expectations. Women are commonly associated with communal roles such as caregiving, nurturing, and emotional support, whereas men are linked to agentic roles involving authority, independence, assertiveness, and leadership (63, 64). As these patterns are repeated across generations, they become normalised and influence education, employment, and leadership selection processes. The theory therefore explains stereotypes as products of social arrangements rather than objective truths about gender.

A key assumption of the theory is that individuals internalise these expectations and institutions often reward behaviour that aligns with dominant role norms. In many workplaces, leadership has historically been associated with decisiveness, control, and competitiveness. Because such traits are more often linked to men, women may encounter obstacles when seeking leadership positions, even when they possess equal qualifications. This helps explain why women are sometimes judged more critically, overlooked for promotion, or required to prove competence repeatedly before advancement opportunities are offered (65, 66).

The relevance of Social Role Theory to leadership advancement studies is significant. It provides a framework for understanding how societal expectations and organisational norms shape promotion outcomes, career mobility, and perceptions of leadership suitability. For women with disabilities, these challenges may be intensified because gender stereotypes can combine with assumptions about disability, competence, and independence. Barriers to advancement may therefore stem not only from inaccessible systems but also from socially constructed beliefs about who is considered capable of leading.

The theory also offers practical value because social roles can be changed. Through policy reform, inclusive leadership development, mentoring, fair promotion systems, and organisational culture change, institutions can reduce bias and expand leadership opportunities (67). For research on leadership advancement among women with disabilities, Social Role Theory provides a strong foundation for analysing how gendered expectations shape professional outcomes and how workplaces can become more equitable and inclusive.

### Integration of the theories

The integration of Intersectionality Theory and Social Role Theory provides a stronger conceptual foundation for this study, as each theory explains a distinct dimension of women with disabilities’ experiences in leadership contexts. When combined, the theories allow the study to examine both structural inequalities and social expectations that shape advancement opportunities. This integrated approach is important because barriers to leadership are rarely caused by a single factor; they often emerge from the interaction of identity, power, stereotypes, and organisational practices (13, 36).

Intersectionality Theory, advanced by Kimberlé Crenshaw, explains how multiple identities, such as gender, disability, race, and class, intersect within systems of power to produce unique experiences of privilege or disadvantage. The theory challenges single-category explanations of discrimination and shows that women with disabilities may experience exclusion in ways that differ from women without disabilities or men with disabilities (30, 31). In this study, the theory is useful for understanding how barriers and enablers are shaped by the combined effects of sexism and ableism in organisational environments.

Social Role Theory, developed by Alice Eagly, explains how socially assigned roles create expectations about behaviour, competence, and leadership suitability. It argues that leadership is often associated with agentic qualities such as authority, assertiveness, and decisiveness, while women are more commonly linked with communal traits such as care and support. These expectations can influence recruitment, promotion, and performance evaluation processes, thereby shaping leadership outcomes (37, 64).

Both theories complement each other because Intersectionality Theory explains *who* is likely to face compounded disadvantage and *why* inequality may be intensified, while Social Role Theory explains *how* stereotypes and social expectations influence organisational decisions. One theory focuses on overlapping systems of oppression, whereas the other focuses on behavioural norms and perceptions of suitability. Together, they provide a more complete explanation of leadership inequality than either theory could provide independently (26, 66).

The theories are suitable for this study because they align directly with the variables under investigation. The independent variable, barriers and enablers, includes factors such as discrimination, inaccessible structures, mentoring opportunities, inclusive policies, and workplace support. Intersectionality Theory helps explain how these factors operate differently for women with disabilities, while Social Role Theory explains how gendered assumptions influence access to these opportunities.

The dependent variable, leadership advancement, is also clarified through the integration of both theories. Advancement is shaped not only by qualifications and performance but also by access to networks, organisational culture, and perceptions of leadership competence. Therefore, the combined framework enables this study to analyse how structural barriers, enabling supports, and gendered expectations interact to shape leadership outcomes for women with disabilities (33, 65).

## Methods

### Review design and reporting

This study employed a PRISMA 2020-guided systematic review with thematic synthesis to synthesise empirical evidence on the barriers and enablers influencing leadership advancement for women with disabilities. The review was conducted and reported in accordance with the Preferred Reporting Items for Systematic Reviews and Meta-Analyses (PRISMA) guidelines (38, 39), ensuring transparency and rigour in the identification, screening, eligibility assessment, and synthesis of relevant studies. The use of PRISMA was appropriate because the study sought to provide a structured, reproducible synthesis of existing evidence rather than an exploratory overview or a narrative summary alone.

A systematic approach was particularly necessary because scholarship at the intersection of gender, disability, and leadership remains fragmented across disciplines such as management, sociology, public health, education, and disability studies. Relevant evidence is dispersed across varied methodological traditions and conceptual perspectives, making a transparent and organised review process essential for identifying recurring patterns and research gaps.

Given the predominance of qualitative, mixed-methods, and interpretive studies in this field, the review prioritised thematic synthesis rather than quantitative meta-analysis (40, 41). A meta-analytic approach was not considered suitable because the included studies varied substantially in design, context, populations, and conceptualisations of leadership advancement. Thematic synthesis enabled the integration of diverse forms of evidence while preserving contextual nuance and conceptual depth.

Accordingly, the review is positioned as a systematic review employing qualitative thematic synthesis to integrate heterogeneous evidence relating to leadership advancement for women with disabilities. This framing reflects the heterogeneous evidence base and the study's aim of generating higher-order analytical insights into how structural, organisational, and identity-related factors shape leadership opportunities for women with disabilities.

### Data sources and search strategy

The literature search was conducted in Scopus, which was selected for its extensive indexing of peer-reviewed journals across social sciences, health, policy, and management. To strengthen coverage and reduce database bias, supplementary searches were conducted on the Web of Science and Google Scholar (42).

The selection of databases was informed by the topic's interdisciplinary scope. While databases such as MEDLINE and EMBASE are widely used in clinical and biomedical reviews, they were not prioritised in this study due to their primary focus on medical and clinical research. Given that the present review addresses leadership, governance, and organisational dynamics within social and policy contexts, Scopus and Web of Science were considered more appropriate for capturing relevant scholarship. This approach is consistent with recommendations for database selection in interdisciplinary social science reviews, which suggest that broader indexing platforms may provide greater conceptual coverage.

Search terms were developed iteratively to capture studies addressing leadership and advancement through gendered and disability-informed lenses. In response to reviewer feedback, the search strategy was expanded to explicitly capture barriers, enablers, and experiential dimensions of leadership. Keyword combinations included variations of women/female, disability/disabled, and leadership, governance, management, decision-making, promotion, or career advancement, alongside terms such as barrier, challenge, discrimination, bias, inequity, enabler, facilitator, support, inclusion, accessibility, experience*, perception*, and perspective.

Searches were applied to titles, abstracts, and keywords. Full search strings for each database are provided in Supplementary Material 1 to enhance transparency and reproducibility.

The search was limited to English-language, peer-reviewed journal articles published between 2021 and 2025, reflecting contemporary debates on leadership, equity, and inclusion. This temporal restriction was applied to capture post-pandemic structural shifts in organisational practices, digital work environments, and policy discourse, which have significantly reshaped leadership pathways and access dynamics.

Google Scholar was used as a supplementary search tool to identify additional relevant studies not indexed in the primary databases. Screening was limited to the first 200 results sorted by relevance, in line with established methodological guidance to balance comprehensiveness and feasibility.

### Eligibility criteria

Studies were included if they:

1. Focused on women with disabilities or examined gender and disability intersectionally;
2. Addressed leadership advancement, governance participation, decision-making, power, or influence as a primary or explicit analytical focus;
3. Reported empirical findings or constituted systematic, scoping, or realist reviews with extractable evidence;
4. Were published in peer-reviewed journals.

Studies were excluded if they focused solely on disability without gendered analysis; examined employment, wellbeing, or participation without leadership relevance; addressed clinical, educational, caregiving interventions unrelated to leadership pathways; or were conceptual papers, editorials, or grey literature.

### Study selection

The search identified 338 records. After removal of 38 duplicates, 300 records were screened by title and abstract, leading to the exclusion of 237 articles that lacked a leadership focus, omitted gender–disability intersectional analysis, or addressed employment, wellbeing, or service delivery without advancement relevance.

**Figure 1:**
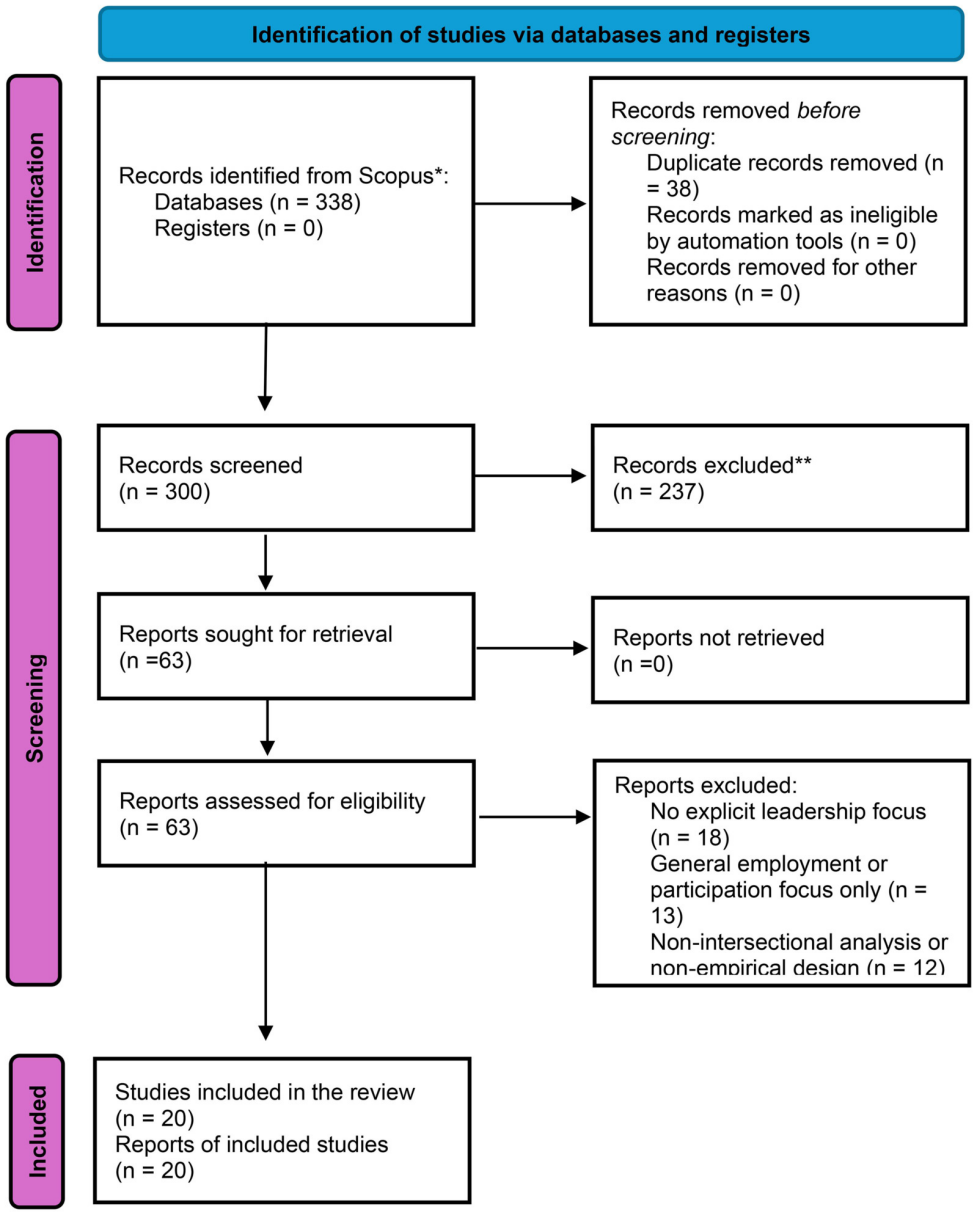
PRISMA 2020 flow diagram of the study selection process.

Screening was conducted independently by two reviewers using the Rayyan software to enhance transparency and reduce selection bias (43). Sixty-three full-text articles were assessed, and 43 were excluded due to the absence of leadership analysis (n = 18), focus on general employment (*n* = 13), non-intersectional treatment (*n* = 7), or non-empirical design (*n* = 5). Disagreements between reviewers were resolved through discussion, and where necessary, consultation with a third reviewer, ensuring consistency in inclusion decisions. Twenty studies were included in the final synthesis. Figure 1 presents the PRISMA 2020 flow diagram of the study selection process.

### Data extraction

Data were extracted using a structured template that captured the author(s) and year; the geographic and sectoral context; the study design; the conceptualisation of leadership; and key findings on the barriers and enablers of leadership advancement. Data extraction was conducted independently by two reviewers using a piloted extraction template, which was refined following initial testing to ensure clarity and consistency. Extraction prioritised analytical findings over descriptive background information, with emphasis on identifying patterns relevant to leadership advancement pathways.

### Analytical synthesis

A thematic synthesis approach was applied (40). Findings were inductively coded, organised into descriptive themes, and refined into higher-order analytical themes (41). Themes were examined across four interrelated levels: structural/institutional, organisational, interpersonal/cultural, and individual/identity-related, enabling analysis of leadership advancement as a multi-level process. Analytical significance was determined by combining the frequency with which themes appeared across studies and the depth of conceptual insight in individual studies.

Conceptual depth was assessed consistently across all included studies using four criteria: the extent to which findings moved beyond description to interpretation; engagement with relevant theory or conceptual frameworks; clarity in identifying underlying mechanisms or causal processes; and the richness of explanation for how barriers or enablers shaped leadership advancement. Studies demonstrating stronger conceptual depth contributed more substantially to the refinement of higher-order themes, while studies offering primarily descriptive findings were used to support pattern identification with more cautious interpretive weighting. This approach enhanced transparency and consistency in determining the analytical contribution of each study.

### Quality and rigour

Methodological quality was appraised based on the clarity of study aims, appropriateness of the research design, transparency of analytical procedures, and coherence between the data presented and the claims made by the authors (68). A CASP-informed appraisal framework adapted for qualitative and mixed-methods studies was applied to ensure relevance to the diverse evidence base included in the review. Quality appraisal was conducted independently by two reviewers, with disagreements resolved through discussion to strengthen consistency and reduce subjective bias.

Studies were not excluded solely because of quality scores or rigid thresholds; conceptual relevance and their contribution to the review questions were also considered important. Instead, critical appraisal informed the interpretation and weighting of evidence within the synthesis. Greater interpretive emphasis was given to studies demonstrating stronger methodological rigour, particularly those with transparent analytical procedures, rich supporting data, and clearly justified conclusions. Studies with less detailed methodological detail were retained where relevant but interpreted with greater caution. Reflexive memos were maintained throughout the review process to enhance analytical transparency, consistency, and rigour.

### Bias considerations

Formal risk-of-bias appraisal was not undertaken, given the qualitative and heterogeneous nature of the included studies and the focus on thematic synthesis rather than effect estimation. Potential selection bias arises from restricting inclusion to English-language, peer-reviewed articles. Although additional databases were consulted, limitations in these databases may still affect coverage.

Publication bias and methodological heterogeneity, particularly the predominance of qualitative studies, may limit transferability. These risks were addressed through transparent eligibility criteria, systematic screening, and emphasis on conceptual rather than effect-based synthesis.

### Limitations

The review was limited to English-language, peer-reviewed literature, potentially excluding relevant studies in other languages or formats. The relatively small number of leadership-focused studies reflects broader gaps in the field. Despite these limitations, the systematic design, expanded search strategy, and theoretically informed synthesis strengthen the credibility and interpretive depth of the review.

## Results

### Study characteristics and context

 Table 1 presents the characteristics of the 20 included studies, detailing their contexts, aims, methodological designs, populations, analytical foci, and relevance to leadership advancement for women with disabilities. The included studies span multiple geographical contexts, including the United States, Canada, Australia, Sweden, Norway, Iceland, Malaysia, and selected African countries. The evidence base is concentrated in high-income contexts, with fewer studies from low- and middle-income settings.

**Table 1:** Extracted data from studies relevant to barriers and enablers to leadership advancement for women with disabilities.

Authors (Year)	Context	Aim/Research Question	Study Design	A	Focus	Key Results (Summarised)	Relevance to Review
Johnson et al. (44)	Health equity, USA	To examine systemic barriers affecting Black people with IDD	Critical participatory action research	Black people with IDD, care partners, providers	Power sharing, co-creation, systemic inequities	Revealed structural barriers, epistemic exclusion, and the need for equitable power redistribution	Direct – Intersectional barriers to power and voice
Refol et al. (52)	Public health governance, Canada	To incorporate equity-owed community perspectives into guidelines	Qualitative	People with disabilities and other equity-owed groups	Inclusion, accessibility, participation	Identified discrimination, accessibility gaps, and the need for participatory governance	Direct – Inclusion in decision-making structures
Riches et al. (51)	Disability policy (NDIS), Australia	To assess choice, control, and well-being	Longitudinal survey	Adults with intellectual disability	Autonomy, decision-making	Limited control over key life decisions despite individualised funding	Direct – Constraints on agency undermine leadership pathways
Reeves et al. (45)	Disability Services, Canada	To explore how belonging is supported	Ethnography	Adults with ID and service leaders	Belonging, rights, relational autonomy	Inclusion requires redistribution of resources and reflexive organisational cultures	Direct – Foundations for leadership inclusion
Best et al. (53)	Disability law, USA	To assess outcomes of discrimination claims	Quantitative legal analysis	Federal court cases	Legal recognition, bias	Mental illness and invisible disabilities face systemic disadvantage	**Direct** – Structural/legal barriers to advancement
Curryer et al. (46)	Organisational governance, Australia	To identify inclusive governance models	Qualitative phenomenology	Board members with and without ID	Leadership, decision-making	Accessible practices and tailored supports enable governance participation	**Direct** – Explicit leadership advancement
.Lombard et al. (49)	Organisational power, USA	To test the effects of feedback receptivity	Experimental (7 studies)	Marginalised & non-marginalised adults	Relational leadership, bias	Feedback-receptive leaders reduce disability and gender bias concerns	**Direct** – Leadership behaviour as enabler
Raišienė et al. (50)	Workplace inclusion, Lithuania	To examine inclusive leadership and work mattering	Cross-sectional survey	Employees with disabilities	Inclusive leadership	Inclusive leadership increases sense of mattering	**Direct** – Leadership culture and advancement
Mansilla et al. (54)	Mobility & access, Canada	To examine perceived transport accessibility	Mixed methods	Adults with mobility disabilities	Accessibility, independence	Social and attitudinal barriers restrict participation	Direct – Structural barriers to professional engagement
Luthra et al. (55)	Employment pathways, Sweden	To explore transitions from daily activity to work	Qualitative	Adults with intellectual disability	Occupation, autonomy	Systemic barriers limit meaningful employment progression	Direct – Career advancement constraints
Mogensen et al. (56)	School-to-adult transition, Australia	To explore post-school life experiences	Co-designed qualitative	Young people with ID	Decision-making, services	Exclusion from planning limits future opportunities	Direct – Pipeline to leadership disrupted
Osterud et al. (57)	Work–family conflict, Norway	To examine career impacts on mothers of disabled children	Qualitative case studies	Mothers of disabled children	Care burden, employment	Inadequate support restricts career advancement	Direct – Gendered leadership barriers
Gjecaj et al. (58)	Access to justice, Iceland	To examine support for disabled women facing GBV	Qualitative	Disabled women, justice workers	Rights protection, advocacy	Knowledge gaps weaken access to justice	Direct – Institutional enablers for women with disabilities
Adenan et al. (59)	Employment inclusion, Malaysia	To explore perceptions of employment	Qualitative	People with Down syndrome, families, and employers	Employment benefits	Employment enhances self-worth and social status	Direct – Economic participation as a leadership foundation
Svanelöv et al. (47)	Disability discourse, Sweden	To analyse participation narratives	Discourse analysis	Support staff & leaders	Ableism, participation	Discourses frame ID as incompatible with leadership	**Direct** – Cultural barriers
Björnsdóttir et al. (60)	Digital inclusion, Iceland	To explore digital exclusion	Mixed-methods	Adults with ID	Digital access, autonomy	Paternalism and lack of support drive exclusion	**Direct** – Digital barriers to leadership
Piano et al. (61)	Academia, Global	To review inclusion challenges for early-career academics	Rapid review	Health academics	Career advancement	Job insecurity and exclusion dominate; disability absent	**Direct** – Reveals systemic omission
Singh (48)	Autism services, USA	Intersectional analysis of service inequities	Narrative analysis	Black single female caregivers	Race, gender, disability	Interlocking systems devalue women's leadership voices	**Direct** – Intersectional leadership barriers
Hultman et al. (62)	Transition to adulthood, Sweden	To explore subject formation	Qualitative	Disabled girls with personal assistants	Identity, authority	Managing assistants positions girls as leaders while constraining autonomy	**Direct** – Leadership paradox

Methodologically, qualitative approaches predominated, including critical participatory action research (44), ethnography (45), phenomenology (46), discourse analysis (47), and narrative analysis (48). Quantitative and mixed-methods studies were also included, such as experimental designs examining leadership behaviour (49), cross-sectional surveys on inclusive leadership (50), and longitudinal analyses of autonomy and decision-making (51).

The populations represented included women with disabilities, adults with intellectual, physical, and mobility disabilities, disabled girls and young women, caregivers, and institutional actors such as service providers, board members, and justice system professionals. Several studies explicitly adopted an intersectional lens to examine how disability interacts with gender, race, caregiving roles, and socioeconomic status (44, 48, 57).

Across the studies, leadership advancement was examined through multiple domains, including governance participation (46, 52), decision-making and autonomy (51, 55), organisational leadership practices (49, 50), and structural access conditions such as legal recognition, mobility, and digital inclusion (53, 54, 60).

### Meta-themes across included studies

The thematic synthesis identified five meta-themes representing the dominant patterns of barriers and enablers influencing leadership advancement for women with disabilities. Table 2 summarises these themes and their frequency across the included studies.

**Table 2:** Meta-themes and frequency of evidence across included studies.

Representative Studies	Meta-Theme	Description of Meta-Theme	Number of Studies (Frequency)
Best et al. (53), Björnsdóttir et al. (60), Curryer et al. (46), Johnson et al. (44), Mansilla et al. (54), Mogensen et al. (56)	**Structural & Institutional Barriers to Leadership**	Barriers embedded in governance systems, legal frameworks, policy design, service structures, and physical/digital environments that restrict access to decision-making and leadership roles	**12**/**20**
Best et al. (53), Lombard et al. (49), Piano et al. (61), Singh (48), Svanelöv et al. (47)	**Ableism, Bias, and Legitimacy Deficits**	Cultural norms and discourses that question the competence, authority, or credibility of women with disabilities, including stigma toward invisible disabilities and deficit framings of leadership	**7**/**20**
Luthra et al. (55), Reeves et al. (45), Riches et al. (51)	**Autonomy, Agency, and Decision-Making Control**	Constraints or support related to exercising choice, control, and self-determination in everyday life, work, and governance as foundations for leadership development	**6**/**20**
Curryer et al. (46), Lombard et al. (49), Raišienė et al. (50), Reeves et al. (45)	**Inclusive Leadership Practices and Organisational Culture**	Leadership behaviours, relational practices, and organisational climates that enable belonging, mattering, psychological safety, and shared power	**7**/**20**

Structural and institutional barriers were the most frequently reported, identified in 12 of the 20 studies. Ableism, bias, and legitimacy deficits were reported in 7 studies. Inclusive leadership practices and organisational culture were identified in 7 studies, while intersectionality and gendered life-course constraints were also identified in 7 studies. Autonomy, agency, and decision-making control were reported in 6 studies.

### Structural and institutional barriers to leadership

Structural and institutional barriers were reported across 12 studies. These barriers were embedded in governance systems, legal frameworks, policy design, and service structures.

Studies described limited inclusion of people with disabilities in decision-making processes and governance structures, as well as restricted access to leadership roles within formal institutions (46, 52). Legal and institutional analyses also identified systemic disadvantages associated with disability status, including inequitable treatment within legal systems and limited recognition of leadership capacity (53).

Barriers related to accessibility were also reported. These included physical and transport-related constraints affecting participation (54), as well as digital exclusion linked to limited support and paternalistic practices (60). Across employment and service systems, studies reported fragmented institutional arrangements and limited opportunities for progression beyond entry-level participation (55, 56).

### Ableism, bias, and legitimacy deficits

Seven studies reported barriers related to ableism, bias, and legitimacy. These studies identified cultural and organisational norms that question the competence, authority, and leadership suitability of individuals with disabilities. Disability was frequently positioned as incompatible with leadership roles, particularly within organisational and professional contexts (47).

Studies also reported stigma and credibility challenges, including differential treatment and reduced recognition of leadership potential, especially for individuals with invisible or psychosocial disabilities (48, 53). These findings also reflected broader patterns of epistemic exclusion, where the perspectives and contributions of individuals with disabilities were undervalued or excluded from decision-making processes (44).

### Autonomy, agency, and decision-making control

Six studies reported findings related to autonomy, agency, and decision-making control. These studies focused on the extent to which individuals with disabilities could exercise choice and influence in personal, organisational, and governance contexts.

Several studies identified constraints on decision-making authority and limited control over key life and work-related choices (51, 55). In transition-related contexts, exclusion from planning processes was reported to limit future opportunities and participation (56). In contrast, some studies described contexts in which supported decision-making and relational autonomy facilitated participation and engagement in leadership roles (45).

### Inclusive leadership practices and organisational culture

Seven studies identified inclusive leadership practices and organisational culture as enabling conditions. These studies described leadership behaviours, organisational environments, and relational practices that support inclusion.

Inclusive leadership behaviours, including feedback receptivity and responsiveness, were associated with reduced perceptions of bias and improved relational dynamics (49). Organisational cultures that emphasised belonging, mattering, and shared decision-making were also associated with increased participation and engagement (45, 50). Studies of governance contexts further identified the role of accessible practices and tailored supports in enabling participation in leadership and decision-making structures (46).

### Intersectionality and gendered life-course constraints

Seven studies identified intersectional and life-course-related constraints. These studies examined how disability interacts with gender, race, caregiving responsibilities, and socioeconomic status across different stages of life.

Studies reported that caregiving roles and gendered expectations affected career progression and access to leadership opportunities (57). Intersectional analyses also identified compounded disadvantage for individuals positioned across multiple marginalised identities, affecting access to resources, recognition, and participation (44, 48).

Evidence from developmental and transition contexts indicated that leadership-related opportunities are shaped early, with constraints in education, service systems, and social participation influencing later access to leadership pathways (56, 62).

## Discussion

### Overview of findings

This review synthesised evidence on the barriers and enablers influencing leadership advancement for women with disabilities across diverse contexts. The findings indicate that leadership advancement is shaped by interactions among structural, organisational, interpersonal, and life-course conditions rather than by individual capability alone (44, 45, 48, 50, 56). Across the studies included, barriers were more frequently reported than enabling conditions, although both were consistently present. Given the relatively small and heterogeneous evidence base, these patterns should be interpreted as indicative rather than definitive.

### Structural constraints and leadership exclusion

Structural and institutional barriers emerged as the most dominant constraint across the evidence base (44, 46, 51–60). These barriers were embedded within governance systems, legal frameworks, service structures, and accessibility conditions that regulate participation in decision-making spaces. This finding aligns with existing research that conceptualises leadership inequality as structurally produced and institutionally maintained rather than individually determined (7, 10).

However, the present review suggests that structural exclusion may operate across multiple stages of engagement, including access to education, employment progression, and governance participation, rather than only at the point of leadership entry (51, 55, 56, 62). These patterns indicate that leadership disparities may be cumulative, reflecting trajectories of constrained opportunities rather than isolated barriers at senior levels, although this interpretation should be considered cautiously given the limited and heterogeneous evidence base.

### Theoretical interpretation of structural constraints

From the perspective of Intersectionality Theory, the structural barriers identified in this review reflect the interaction of multiple systems of oppression, particularly sexism and ableism, operating within organisational and institutional settings (13, 14). Rather than functioning as isolated obstacles, inaccessible governance systems, weak policy implementation, limited accommodations, and exclusion from decision-making spaces demonstrate how institutions can normalise unequal access to leadership opportunities (46, 53). Women with disabilities may therefore encounter disadvantages that differ from those experienced by women without disabilities or men with disabilities because their marginalisation is shaped by overlapping identities and power relations. The findings further suggest that formal inclusion policies are insufficient when organisational practices and accountability systems fail to challenge embedded inequalities (45, 52). This extends existing leadership literature by showing that advancement barriers are not only interpersonal or attitudinal but are also structurally reproduced through systems that determine participation, visibility, and progression. Consequently, improving leadership inclusion requires institutional redesign, accessibility reform, and equity-focused implementation rather than relying solely on individual resilience. Ableism, Legitimacy, and Leadership Norms.

The findings relating to ableism and legitimacy deficits indicate that leadership exclusion is also shaped by cultural and organisational expectations. Across the studies, disability was frequently constructed as incompatible with leadership, resulting in reduced credibility, heightened scrutiny, and restricted access to authority (47, 48, 53).

This pattern can be interpreted through Social Role Theory, which posits that leadership is associated with socially constructed norms of competence, independence, and authority (69). When disability is perceived as deviating from these expectations, individuals may be excluded from leadership consideration, regardless of their capabilities. Similar patterns have been reported in leadership research, where deviations from normative leadership prototypes affect evaluation and advancement (11). Within this framework, legitimacy can be understood as socially negotiated rather than fixed, shaped by prevailing norms and expectations.

### Autonomy and agency as foundations for leadership

The findings demonstrate that autonomy, agency, and decision-making control are foundational to leadership development. Constraints on everyday decision-making, access to resources, and participation were associated with limited opportunities to develop leadership-related skills and confidence (51, 55, 56). From an intersectional perspective, these constraints are shaped by overlapping social identities, including gender, disability, caregiving responsibilities, and socioeconomic status (13, 14).

Importantly, the findings suggest that leadership trajectories may be influenced by early experiences of agency across life domains, including education, service systems, and social participation. This interpretation supports the possibility that leadership development is cumulative and path-dependent, although stronger longitudinal evidence is needed to confirm these relationships.

### Enabling conditions: inclusive leadership and organisational culture

Although barriers were prominent, the findings also identified enabling conditions that support leadership participation and progression. Inclusive leadership practices, organisational cultures of belonging, and relational approaches to decision-making were consistently associated with increased participation and engagement (45, 46, 49, 50). These findings align with literature on inclusive leadership, which emphasises responsiveness, trust, and shared power as key mechanisms for inclusion. However, enabling conditions may be necessary but not sufficient in the absence of broader structural change.

### Intersectionality and life-course dynamics

The findings further indicate that leadership exclusion may unfold across the life course, with early constraints in participation and autonomy potentially shaping later access to leadership pathways (55, 56, 62). This interpretation is consistent with life-course and intersectionality frameworks, though conclusions should remain cautious given the scope and composition of the included studies.

### Contribution of the review

This review makes several contributions to the literature. First, it integrates fragmented evidence across disciplines to provide a multi-level understanding of the advancement of women with disabilities in leadership. Second, it demonstrates that leadership exclusion is shaped by structural, cultural, and relational dynamics. **Third, and most significantly, the review suggests that leadership pathways may be cumulative, with early constraints in autonomy, participation, and access potentially influencing later leadership opportunities. This shifts attention from leadership positions alone to leadership trajectories, while recognising the need for further evidence across diverse contexts.**

### Research gaps and future directions

Despite the growing body of evidence, several gaps remain. Literature is heavily concentrated in high-income contexts, with limited representation from the Global South. There is also a lack of longitudinal research examining how leadership pathways develop over time and limited attention to sector-specific dynamics, particularly in political, corporate, and community leadership contexts. Future research should prioritise longitudinal and contextually diverse designs to better capture how leadership trajectories evolve across different socio-political settings.

### Limitations of the study

This review has several limitations that should be acknowledged. First, the review was restricted to English-language, peer-reviewed studies, which may have excluded relevant evidence published in other languages or grey literature. Second, the relatively small and heterogeneous evidence base limits broad generalisation across all contexts. Third, most included studies were conducted in high-income countries, reducing representation of low- and middle-income settings. Fourth, variations in the conceptualisation of leadership across studies may have affected the consistency of synthesis. Despite these limitations, the systematic design, transparent screening procedures, and theory-informed analysis strengthen the credibility of the review.

## Conclusion

This systematic review synthesised evidence on the barriers and enablers influencing the leadership advancement of women with disabilities across diverse contexts. The findings demonstrate that leadership advancement is shaped by interconnected structural, organisational, interpersonal, and life-course conditions rather than individual capability alone. Structural and institutional barriers remain the most pervasive constraints, while enabling conditions, particularly inclusive leadership practices and autonomy-supportive environments, play a critical role in facilitating participation and progression.

A key contribution of this review lies in demonstrating that leadership pathways are cumulative and shaped over time. Early constraints in autonomy, decision-making, and access to participation limit the development of leadership capacity and influence later opportunities for advancement. This shifts the focus from leadership positions to leadership trajectories, highlighting the need to address barriers across the life course.

The findings have important implications for policy and practice. Efforts to promote leadership inclusion for women with disabilities must extend beyond formal policy commitments to address structural inequalities, accessibility barriers, and organisational cultures that shape participation. Future research should prioritise longitudinal and context-sensitive approaches, with greater attention to underrepresented regions and sectors, to deepen understanding of how enabling conditions can be effectively implemented and sustained.

In addition, the review highlights that inclusive leadership is not solely a matter of representation but also of redistributing power, voice, and decision-making opportunities within organisations and institutions. Women with disabilities should be recognised not as passive beneficiaries of inclusion policies, but as capable leaders whose perspectives strengthen governance, innovation, and organisational effectiveness. This requires intentional investment in accessible leadership pipelines, mentoring structures, sponsorship opportunities, and fair promotion systems. The review also underscores the importance of moving from symbolic inclusion to measurable accountability, where organisations evaluate progress through transparent indicators of equity and advancement. While the evidence base remains emergent, the consistency of themes across studies suggests that meaningful change is achievable when structural reform is combined with cultural transformation. Advancing the leadership of women with disabilities is therefore both a social justice imperative and a strategic pathway toward more diverse, responsive, and equitable leadership systems.

## Data Availability

The original contributions presented in the study are included in the article/Supplementary Material, further inquiries can be directed to the corresponding author.
