# Supplementary material for: Barriers and enablers to leadership advancement for women with disabilities: a systematic literature review
Source: Front Rehabil Sci. 2026 Jun 15;7:1813535. doi: 10.3389/fresc.2026.1813535 (PMC13310883; doi:10.3389/fresc.2026.1813535)
Supplement: Supplementary file 1 [file Datasheet1.pdf]

Supplementary Material 1: Full Search Strategy

Supplementary Material 1. Search Strategy and Database Queries

To enhance transparency and reproducibility, the full search strategies used across databases are provided below. Search terms were developed iteratively to capture literature on leadership advancement at the intersection of gender and disability, including barriers, enablers, and experiential dimensions.

1. Scopus Search String

(TITLE-ABS-KEY (women OR woman OR female OR gender) AND

TITLE-ABS-KEY (disability OR disabilities OR disabled OR impairment) AND

TITLE-ABS-KEY (leadership OR leader OR governance OR management OR "decision making" OR promotion OR "career advancement") AND

TITLE-ABS-KEY (barrier OR challenge OR discrimination OR bias OR inequity OR enabler OR facilitator OR support OR inclusion OR accessibility OR experience* OR perception* OR perspective*))

AND (LIMIT-TO (LANGUAGE, "English"))

AND (LIMIT-TO (DOCTYPE, "ar"))

AND (PUBYEAR > 2020 AND PUBYEAR < 2026)

2. Web of Science Search String

TS = ((women OR woman OR female OR gender) AND

(disability OR disabilities OR disabled OR impairment) AND

(leadership OR leader OR governance OR management OR "decision making" OR promotion OR "career advancement") AND

(barrier OR challenge OR discrimination OR bias OR inequity OR enabler OR facilitator OR support OR inclusion OR accessibility OR experience* OR perception* OR perspective*))

Refined by: Document Types = Article; Languages = English;

Timespan = 2021–2025

3. Google Scholar Search Strategy

Google Scholar was used as a supplementary search tool to identify additional relevant studies not captured in primary databases.

Search String Used:

"women with disabilities" AND leadership AND (barriers OR enablers OR inclusion OR advancement)

Screening Procedure:

- Results were sorted by **relevance**
- **First 200 records** were screened
- Titles and abstracts were reviewed for eligibility
- Duplicate studies already identified in Scopus and Web of Science were removed

4. Search Strategy Development

The search strategy was developed iteratively and refined to ensure sensitivity to:

- Intersectionality (gender + disability)
- Leadership and advancement pathways
- Structural and experiential dimensions (barriers, enablers, perceptions)

Keywords were grouped into four conceptual clusters:

1. **Population:** women, female, gender
2. **Condition:** disability, disabled, impairment
3. **Outcome:** leadership, governance, management, career advancement
4. **Contextual Factors:** barriers, enablers, inclusion, accessibility, experiences

Truncation (*) and Boolean operators (AND, OR) were used to maximise retrieval of relevant studies.

5. Inclusion Limits Applied

- Language: English
- Publication Type: Peer-reviewed journal articles
- Timeframe: 2021–2025

The timeframe was selected to capture recent post-pandemic shifts in organisational practices, leadership structures, and inclusion discourse.

6. Notes on Database Selection

Scopus and Web of Science were selected due to their broad interdisciplinary coverage across social sciences, policy, and organisational research.

Databases such as MEDLINE and EMBASE were not included because their primary focus is clinical and biomedical literature, which was not central to this review's objectives.
